# Risk scorecard to minimize impact of COVID-19 when reopening

**DOI:** 10.1093/jtm/taab113

**Published:** 2021-07-23

**Authors:** Shin B Lim, Rachael Pung, Kellie Tan, Jocelyn H S Lang, Dominique Z X Yong, Shi-Hua Teh, Elizabeth Quah, Yinxiaohe Sun, Stefan Ma, Vernon J M Lee

**Affiliations:** Ministry of Health, Singapore; Ministry of Health, Singapore; London School of Hygiene and Tropical Medicine, UK; Ministry of Health, Singapore; Ministry of Health, Singapore; Ministry of Health, Singapore; Ministry of Health, Singapore; Ministry of Health, Singapore; Saw Swee Hock School of Public Health, National University of Singapore, Singapore; Ministry of Health, Singapore; Saw Swee Hock School of Public Health, National University of Singapore, Singapore; Ministry of Health, Singapore; Saw Swee Hock School of Public Health, National University of Singapore, Singapore

**Keywords:** SARS-CoV-2, social distancing, imported cases, contact tracing, quarantine, traveller testing, infectious diseases

## Abstract

**Background:**

We present a novel approach for exiting coronavirus disease 2019 (COVID-19) lockdowns using a ‘risk scorecard’ to prioritize activities to resume whilst allowing safe reopening.

**Methods:**

We modelled cases generated in the community/week, incorporating parameters for social distancing, contact tracing and imported cases. We set thresholds for cases and analysed the effect of varying parameters. An online tool to facilitate country-specific use including the modification of parameters (https://sshsphdemos.shinyapps.io/covid_riskbudget/) enables visualization of effects of parameter changes and trade-offs. Local outbreak investigation data from Singapore illustrate this.

**Results:**

Setting a threshold of 0.9 mean number of secondary cases arising from a case to keep R < 1, we showed that opening all activities excluding high-risk ones (e.g. nightclubs) allows cases to remain within threshold; while opening high-risk activities would exceed the threshold and result in escalating cases. An 80% reduction in imported cases per week (141 to 29) reduced steady-state cases by 30% (295 to 205). One-off surges in cases (due to superspreading) had no effect on the steady state if the R remains <1. Increasing the effectiveness of contact tracing (probability of a community case being isolated when infectious) by 33% (0.6 to 0.8) reduced cases by 22% (295 to 231). Cases grew exponentially if the product of the mean number of secondary cases arising from a case and (1—probability of case being isolated) was >1.

**Conclusions:**

Countries can utilize a ‘risk scorecard’ to balance relaxations for travel and domestic activity depending on factors that reduce disease impact, including hospital/ICU capacity, contact tracing, quarantine and vaccination. The tool enabled visualization of the combinations of imported cases and activity levels on the case numbers and the trade-offs required. For vaccination, a reduction factor should be applied both for likelihood of an infected case being present and a close contact getting infected.

## Introduction

To halt the spread of coronavirus disease 2019 (COVID-19), many countries imposed a combination of border control measures, stay-home-orders, shut-down of activities and physical distancing, generally referred to as ‘lockdown’ measures. The economic and social costs of these widespread lockdowns have been severe.^1^^,^^2^ The long-term imposition of lockdown measures is not sustainable socially, economically and politically. Many countries exited from their first lockdowns with varying success in sustained control of COVID-19 transmission.^3^ Even countries lauded for their initial effective lockdown policies have met with second waves of infection due to seeding of cases from inbound travel or relaxation of domestic control policies, resulting in the re-imposition of lockdown measures in many countries.^4–8^ Frequent re-imposition of measures leads to substantial economic and social fallout and is undesirable. Lockdowns have given countries time to build up community public health measures, testing capabilities, improve contact tracing abilities and increase healthcare capacity. Used appropriately, these tools can aid countries in exiting lockdowns in a safe and sustainable manner.

The relaxation of lockdown measures requires a delicate balancing act—to allow enough of the economy and society to function whilst ensuring that case numbers are kept within the ability of containment capabilities to prevent runaway epidemics, and within the capacity of the healthcare system, to prevent over-burdening of medical resources that may lead to increased morbidity and mortality. Trade-offs are necessary—as opening up international travel increases the possibility of imported cases though mitigated by the restrictions placed on travellers, and needs to be balanced against the relaxation of restrictions on domestic activity, in order to ensure lower rates of transmission arising from community cases. Trade-offs between various domestic activities also have to be made in order to keep the risk of community transmission and hence case numbers below acceptable limits. At the same time, testing and contact tracing capabilities can reduce the potential spread of community cases, hence allow for a greater degree of social interaction.

We searched PubMed, BioRxiv and MedRxiv for articles published in English from inception to 1 Dec 2020, with the keywords ‘2019-nCoV’, ‘novel coronavirus’, ‘COVID-19’, ‘SARS-CoV-2’ AND ‘testing’, ‘importation risk’, ‘social distancing’, reproduction number’, ‘R0’, ‘transmission’. We found various papers that considered the impact of imported cases, relaxation of social distancing measures and various testing and quarantine regimes on transmission, as independent factors. We have not found any papers that have attempted to study the effects of these various measures in combination.

We propose a novel approach to decision-making for exiting from lockdown using a ‘risk scorecard’. It is premised on the fact that at any point in time, a society and healthcare system can accept a certain level of risk of transmission of cases—within which various economic and social activities can function, beyond which there will be containment and healthcare system collapses, resulting in an uncontrolled surge in cases and ultimately mortalities.

We describe a model to enable the calculation of the effect of imported cases from travellers, and various community-based societal activities on disease transmission and their summation, considering the impact of mitigating measures such as social distancing policies, testing and containment/contact tracing capabilities. With its widely available data on various COVID-19-related parameters, the city-state of Singapore has used this approach to open up its society and economy.

The intent of this tool is to allow policy makers around the world to weigh the relative impact of various activities in their contexts in order to prioritize a basket of activities to resume that remains within the overall risk score. The parameters of the model can be adapted for use in a country based on specific requirements. An online tool has been developed (hosted on https://sshsphdemos.shinyapps.io/covid_riskbudget/) which enables visualization of the effects of various parameter changes on case numbers and trade-offs to be considered.

## Methods

### Determining the contributions of a population’s activities to the budget

For household and workplace activities, we estimated the mean number of secondary cases that would arise should there be a single case in a household/workplace, using observed attack rates and average number of close contacts based on local outbreak investigation data.

For social activities, we estimated the mean number of secondary cases that would arise from a case based on all social activities engaged by the population. This was the sum of the mean number of secondary cases arising from a case based on each activity, which is determined by the baseline probability of infection assuming a case talking to other contact(s), none of whom are wearing any personal protective equipment (PPE), relative risk of the activity, estimated number of close contacts a case would have during that activity and likelihood of a case attending that activity.

**Figure 1:**
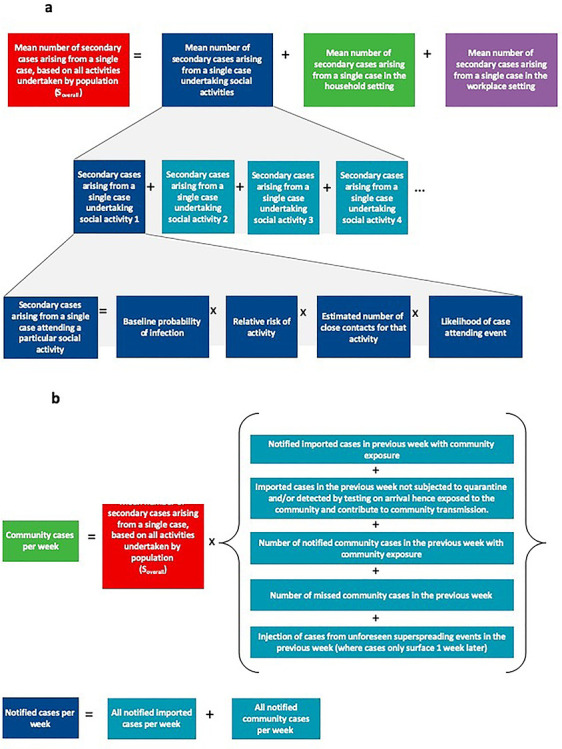
**a:** Calculation of mean number of secondary cases arising from a single case based on all activities undertaken by the population (${S}_{overall})$ **b:** Calculation of notified cases per week.

The overall mean number of secondary cases arising from a case based on all activities undertaken by the population (${S}_{overall})$is thus the sum of the mean number of secondary cases arising from a case based on households, workplaces and activities undertaken by the population. Figure 1a provides an outline of how these values are calculated, with more details in the supplementary appendix.

### Determining the weekly incidence

Given a generation time of COVID-19 of 5.2 days,^9^ the number of cases generated in the community in a week is estimated to be a product of the number of cases in the previous week and the overall mean number of secondary cases arising from a single case based on activities undertaken by the population. Travellers pose a risk by importing cases into the country based on the incidence of COVID-19 in the country of travel origin—however, this can be mitigated by testing and quarantine regimes for travellers to reduce the COVID-19 exposures from travellers into the community. Travel therefore results in imported cases that do not have community exposure and will need to be managed within the healthcare capacity, and those that have community exposure and may contribute to further transmission. Overall, the number of cases in the previous week is contributed by imported cases and community cases and is influenced by traveller quarantine and testing regimes and contact tracing capabilities in the community respectively. Figure 1b provides an outline of how the number of notified cases per week is calculated, with more details in the supplementary appendix.

There will be a proportion of imported/community cases that will not be notified, i.e. they will not be picked up through active COVID testing or by presenting themselves for treatment at healthcare facilities. These cases that are not notified will contribute to community transmission the following week but will not utilize healthcare resources. Only the notified cases will utilize healthcare resources hence this is the number that should be kept within an acceptable level.

To assess the impact of change of various parameters on the number of notified cases per week, we simulated a variety of scenarios using the Singapore population as an example. Singapore is a city-state in Southeast Asia with a population of 5.7 million as of November 2020.^10^ Values used were approximations of the situation in Singapore as of August 2020, after the lifting of control measures in June 2020. The values used in our calculations are summarized in Table 1 below.

**Table 1:** Values used for calculation of notified cases per week

Scenario	Mean number of secondary cases arising from a case based on all activities undertaken by population	Current community cases (Week 1)	Imported cases per week	Probability of an imported case being notified (ratio of imported cases picked up during quarantine: all imported cases)	Probability of a case in the community being isolated (indicating contact tracing efficiency)	Proportion of notified imported cases who are infectious and have exposure to susceptible contacts	Proportion of notified community cases who are infectious and have exposure to susceptible contacts	Injection of cases from unforeseen superspreading events in the previous week (where cases only surface 1 week later)
Baseline	0.9	100	141	0.95	0.6	0.5	0.1	100, each week from week 1–10
*Varying no. of imported cases*								
1	0.9	100	29	0.95	0.6	0.5	0.1	100, each week from week 1–10
2	0.9	100	282	0.95	0.6	0.5	0.1	100, each week from week 1–10
*Sudden injects of additional cases at a specific week to simulate a large cluster from a one-off super-spreading event*								
3	0.9	100	141	0.95	0.6	0.5	0.1	100 (week 1 through 10), additional 30 at week 4
4	0.9	100	141	0.95	0.6	0.5	0.1	100 (week 1 through 10), additional 50 at week 4
5	0.9	100	141	0.95	0.6	0.5	0.1	100 (week 1 through 10), additional 100 at week 4
6	0.9	100	141	0.95	0.6	0.5	0.1	100 (week 1 through 10), additional 100 at week 2
*Varying the likelihood of a community case being isolated (i.e. effectiveness of contact tracing and quarantine)*								
7	0.9	100	141	0.95	0.8	0.5	0.1	100, each week from week 1–10
8	0.9	100	141	0.95	0.4	0.5	0.1	100, each week from week 1–10
*Varying S_overall_ and n_comm_ together*								
9	1.7	100	141	0.95	0.6	0.5	0.1	100, each week from week 1–10
10	2.1	100	141	0.95	0.6	0.5	0.1	100, each week from week 1–10
11	0.9	100	141	0.95	0.4	0.5	0.1	100, each week from week 1–10
12	1.7	100	141	0.95	0.4	0.5	0.1	100, each week from week 1–10
13	2.1	100	141	0.95	0.4	0.5	0.1	100, each week from week 1–10

### Setting a budget

The aim of the risk scorecard is to keep the weekly incidence of notified cases stable and below acceptable limits over time.

Once a threshold level is set for the mean number of secondary cases arising from a case based on all activities undertaken by population, the amount and nature of activities (e.g. event size, requirement for mask-wearing, number of close contacts) can be altered to obtain a combination that allows the mean number of secondary cases to remain below this threshold. For our calculations, we set the threshold at 0.9, to allow for some buffer due to uncertainty in the scale of social interactions so that the mean number of secondary cases does not exceed 1.

Similarly, a threshold number of notified cases per week must be set. Within this threshold, exact quantities of the various parameters can be altered to obtain a number of combinations where the number of notified cases per week is below the threshold. For our calculations, we set the threshold of notified cases per week as 350, where the contact tracing and healthcare system can sustainably cope over long periods of time.

All analyses were performed in *R*^11^.

## Results

### Determining further activities that can be resumed based on the threshold level set for the mean number of secondary cases arising from all activities undertaken by population

With a threshold of 0.9 mean number of secondary cases arising from a case based on all activities undertaken by the population and the household and workplace settings contributing already contributing 0.38 secondary cases per case, we determined that social activities allowed in the population should not contribute more than 0.52 secondary cases per case.

Table 2 shows a worked example of types of activities that are under consideration for reopening. These calculations indicate that opening all activities excluding choirs and nightclubs would result in fewer than 0.52 secondary cases per week per case hence stay within the threshold, whereas opening all activities including choirs and nightclubs would result in more than 0.52 secondary cases per week per case, and hence exceed the threshold.

**Table 2:** Worked example of types of activities that are under consideration for reopening

Event type	Event size	Prob (appear) activity	prob(infection)_baseline_	relative risk_activity_	No. of close contacts	S_activity_	No. of such events per week	S for all such activities	Resume?
General social interactions e.g. meeting friends, going to the restaurant, movies	8	0.0000016	0.02	1	16	0.00000051	625 000	0.32	Yes
Smaller-scale concerts with no talking	5000	0.001	0.02	0.0225	5	0.00000225	30	0.0000675	Yes
Meetings, Incentives, Conferences, Exhibitions	5000	0.001	0.02	0.1125	30	0.0000675	50	0.003375	Yes
Choirs	50	0.00001	0.02	20	20	0.00008	300	0.024	No
Nightclubs	500	0.0001	0.02	20	50	0.002	300	0.6	No
Cultural celebrations and commemorative events	1000	0.0002	0.02	20	50	0.004	15	0.06	Yes
Mass concerts, music festivals and spectator sports with shouting/ loud talking	10 000	0.002	0.02	20	10	0.008	5	0.04	No

### Effect of varying parameters on notified cases per week

**Figure 2:**
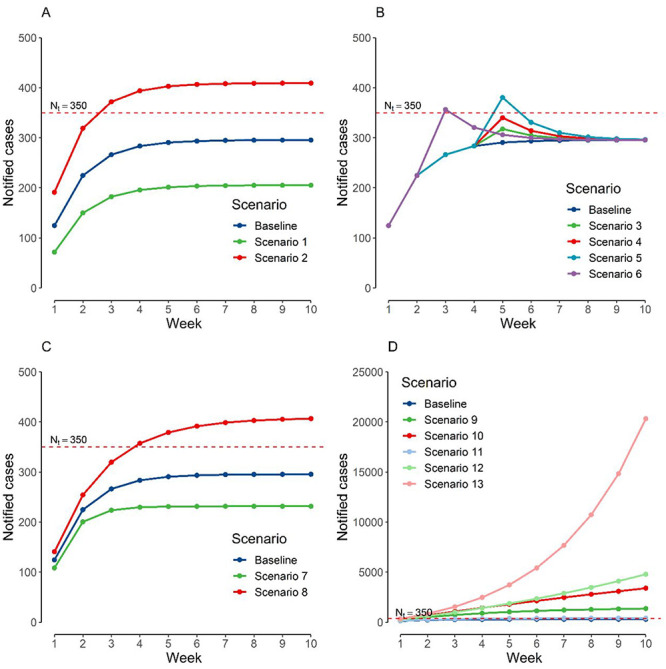
Weekly notified cases based on respective scenarios. (a) Varying number of imported cases per week, (b) one-off surges in cases at respective time points, (c) varying probability of a community case being isolated, (d) varying imported cases and mean number of secondary cases arising from a case based on arising from all activities undertaken by the population on notified cases per week.

Imputing base-case scenario numbers for all parameters resulted in steady-state notified cases per week of 364, which is slightly in excess of the set threshold of 350. The value of current community cases (week 0) did not have an effect on the eventual steady-state notified cases per week$.$ A decrease from 141 to 29 imported cases per week, all else unchanged, resulted in a reduction of steady-state notified cases per week from 295 to 205. An increase to 282 imported cases per week resulted an increase in the steady-state notified cases per week to 409 (Figure 2a).

One-off large surges in case numbers of various numbers of cases at various time points resulted in a short-term surge in notified cases per week above the threshold but had no effect on the steady state (Figure 2b). Increasing the probability of a community case being isolated from 0.6 to 0.8 reduced steady-state NW from 295 to 231. Reducing the probability of a community case being isolated from 0.6 to 0.4 reduced steady-state notified cases per week from 295 to 406 (Figure 2c).

**Figure 3:**
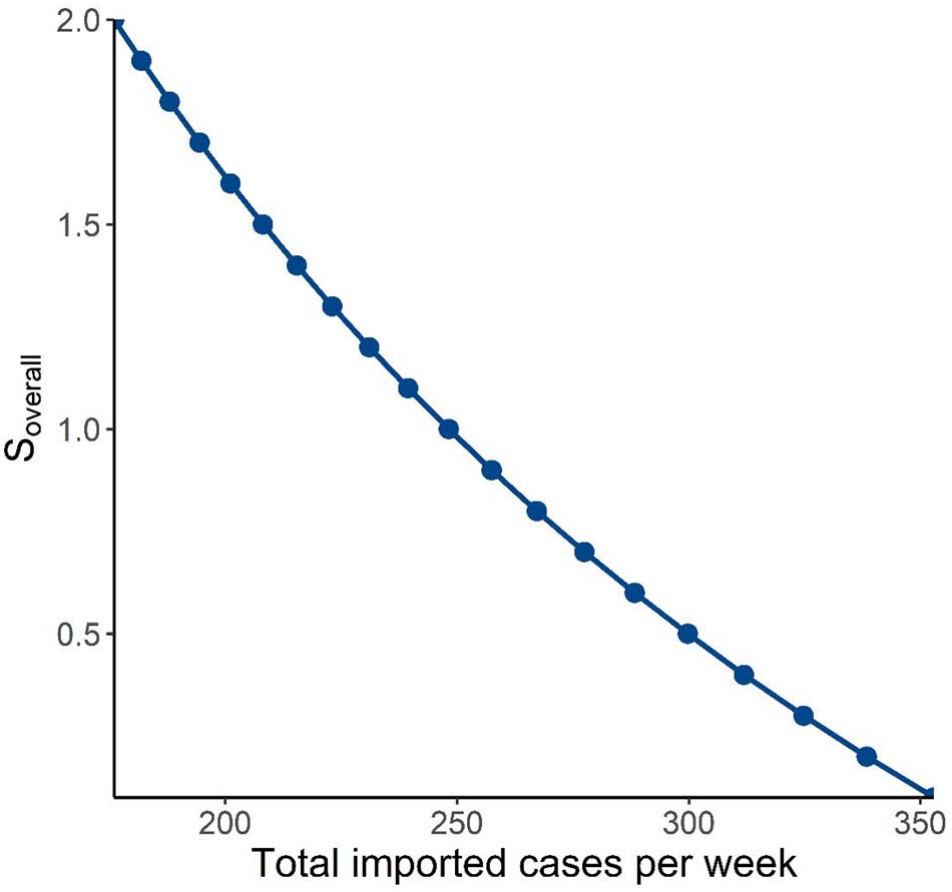
Allowable number of imported cases per week for respective mean number of secondary cases arising from all activities undertaken by the population at a fixed health care capacity of 350 persons per week, based on (i) Probability of an imported case being notified (ratio of imported cases picked up during quarantine: all imported cases) of 0.95; (ii) Probability of a case in the community being isolated (indicating contact tracing efficiency) of 0.6; (iii) Proportion of notified imported cases who are infectious and have exposure to susceptible contacts of 0.5; and (iv) Proportion of notified community cases who are infectious and have exposure to susceptible contacts of 1.

We tested a range of combinations of values for the probability of a community case being isolated and mean number of secondary cases arising from a case based on all activities undertaken by the population to consider the joint effects of contact tracing and quarantine capabilities and changes in community activity levels. An exponential growth of notified cases week-on-week will result if the product of the mean number of secondary cases arising from a case based on all activities undertaken by the population and (1—probability of a community case being isolated) is >1 (Figure 2d). Finally, we plotted the values of imported cases per week and mean number of secondary cases arising from a case based on all activities undertaken by the population that would result in the a steady-state number of notified cases within the threshold, keeping all other variables constant at baseline values. Any combination of imported cases per week and mean number of secondary cases arising from a case based on all activities undertaken by the population that is below the curve will result in notified cases staying within the threshold (Figure 3).

## Discussion

The risk scorecard presents a conceptual framework and quantitative way of mapping out the complex interactions between the various components of transmission risk and mitigating factors that affect case numbers within a population. It allows easy visualization of the various policy decisions and trade-offs that have to be made in order to keep case numbers within a defined threshold. Values of the parameters can be changed to reflect the situation in respective countries or improvements in the effectiveness of mitigation measures based on research developments. Some countries have high prevalence of recovered and presumably immune individuals, and with the introduction of vaccines the level of immunity could be even higher through vaccination programs. The immunity level and the corresponding reduction in risk of infection can easily be included in the model to determine the risk score allowable under these new parameters. This would also allow policy makers to perform forward planning on what vaccinations could do for opening up society and the economy, and should be explored in future research studies.

In deciding which activities to resume, trade-offs have to be made based on socio-economic-political considerations. For example, activities that score highly on S_activity_, hence take up a significant proportion of S_overall_, are those with (i) prolonged droplet and aerosol generation (singing and loud talking), (ii) lack of mask usage, and (iii) a large number of contacts. Such activities include karaokes, nightclubs and bars and religious congregational activities, all of which have resulted in a large number of cases from a single/few positive case(s) attending these events.^11–13^ These activities are also inherently social in nature and safe distancing is difficult to practice. While they are generally of low economic value, they may have a substantial social or political lobby. While these activities in their pre-COVID-19 form have a high risk score, the nature of these activities can materially altered to reduce transmission in these settings. Conversely, events where the audience is seated with physical distancing, wearing masks, and do not generally interact have low S_activity_. Many of these have also have high economic or social value and may be prioritized to safely proceed.

Using the worked example of Singapore, where this approach has been successfully implemented, several conclusions could be drawn. Firstly, in systems where each infected individual spreads to less than one person (i.e. reproduction number, R < 1), imported cases constitute ‘injects’ that may lead to further generations of community transmission, though overall case numbers will eventually drop to steady state, even in the face of continuing imported cases. The number of imported cases depends on the number of inbound travellers and the community incidence rates in the countries of origin. Effective travel restrictions, traveller quarantine and testing policies will reduce the number of infectious imported cases that interact with the community and result in further generations of cases.

In particular, countries that serve as major air transportation hubs are prone to disease importation and subsequent large-scale community transmission, including new Variants of Concern. Prompt imposition of restrictions on air travel when there is local transmission from travellers in the destination country was shown to be effective in reducing spread.^14^ For incoming travellers, quarantine for the incubation period and testing upon arrival and before release will reduce community transmission. A study^15^ found that 79.6% of infected travellers are infectious upon arrival; screening with a 14-day isolation of test-positives followed by a negative test achieves a 91.7% reduction in secondary cases, versus only a 55.4% reduction for no screening but a 7-day mandatory quarantine. The degree of reduction can be further modified by altering the risk profile of travellers entering. Restricting arrivals to countries with low incidence rates or to vaccinated individuals, or imposing a quota on travellers from countries of higher risk or unvaccinated individuals, has been effective in preventing a surge in cases in the receiving country^16^; however this policy needs to be frequently reviewed in response to epidemic trends and vaccine effectiveness.

It is noted that both imported cases that enter the community and those that are detected during quarantine will exert pressure on healthcare resources. Countries should also consider imposing requirements for pre-departure testing in order to reduce the healthcare impact of imported cases. The current utility of an ‘immunity certificate’ where a traveller can be documented as having recovered from COVID-19 or been vaccinated is unclear, as that is insufficient to address several concerns,^17^ including the level and durability of immunity and whether they preclude shedding of transmissible virus, whether antibody tests are required for verification. Nevertheless, recovered and/or vaccinated travellers could have certain exemptions, e.g. reductions to the duration of quarantine, if the assessed risk is similar to a COVID-19-naïve traveller without exemptions.

The effect of seeding by imported cases on a background of lax social distancing policies (and where R > 1) should not be discounted—in Singapore, imported cases from returning residents in March seeded an outbreak among foreign workers and led to over 50 000 PCR-confirmed cases in foreign worker dormitories.^18^ Countries that are considering relaxing travel restrictions to allow more travellers to enter should therefore consider maintaining social distancing measures (including mask mandates) in order to keep community transmission risks low (i.e. maintain R < 1) and case numbers within manageable levels.

A good system of containment measures including contact tracing, testing and quarantine operations is an enabler for allowing a greater range of activities to resume. The widespread deployment of digital contact tracing tools may play a key role in improve the speed and completeness of quarantine of close contacts and therefore remove cases from circulating the community.^19–22^ The proportion of first-generation community cases remaining in the community (a function of the effectiveness of containment measures) and the number of second-generation cases arising from all activities undertaken by a particular case are inversely correlated at a given threshold of new cases per week, and the multiple of these two parameters should not exceed 1—otherwise, exponential increase in cases will occur. Even the best contact tracing system is unlikely to detect and remove 100% of contacts and cases from circulation in the community; therefore it would be prudent to maintain some degree of limitations on social activities compared to the pre-COVID-19 era.

Having effective containment measures coupled with calibrated opening of social activities also allows the population to withstand the impact of one-off superspreading events that may produce large numbers of first-generation cases. All else unchanged, even after such one-off superspreading events, the number of new cases per week will eventually return to a steady-state, while the healthcare system may be placed under strain for a period of time, necessitating surge capacity to be in place. Therefore, it would be prudent to include a buffer in the risk scorecard to account for secondary unknown and unanticipated effects of further relaxations (e.g. a primary activity creating opportunities for further interaction), ongoing higher risk activities that are not known to governments, and reaction time for unexpected events.

Community-based testing is another tool that will need to be considered, as testing as part of surveillance or active case-finding will help to identify cases for early isolation and contact tracing. Pre-event testing can also be used to reduce the probability of an infectious COVID-19 case entering an event and reduces the overall risk of secondary spread for any given activity, all else being equal. Conversely, reduction of risk from pre-event testing would also mean that the number of people participating in any activity could increase while maintaining the overall activity budget.

There are several limitations to this study. This is a simplified model that supports policy making and is easy to use, but does not account for behavioural changes across time, which may occur in response to changes in policies or fatigue from prolonged imposition of social distancing measures resulting in decreasing compliance. We have also assumed that the disease parameters are fixed over a 7-day timeframe, and that the following generation of cases will be captured exactly after 7 days. We have made the model code available on https://sshsphdemos.shinyapps.io/covid_riskbudget/, which can be modified by the end-user. Finally, the accuracy of outcomes is highly dependent on the quality of input data. This may be a challenge in countries where there are limited data on COVID-19 transmission or an inability to control or predict the population’s activities. Nevertheless, the simplicity of the model allows parameters to be changed when new data become available for dynamic policy making.

## Conclusion

The risk scorecard enables easy visualization of the relative effects of various societal activities and mitigation measures on COVID-19 case numbers. It shows that it is possible for a society to resume a wide range of activities for majority of the population whilst keeping the risks of an outbreak low. These are generally activities where masks are worn and there is limited talking and aerosol generation. For higher risk activities, resumption should be coupled with mitigating measures such as frequent testing of patrons/staff, vaccination and improved ventilation of indoor venues.

In opening travel, countries should take a risk-based approach. The number of imported cases can be managed upstream by imposing pre-departure testing/vaccination requirements and restrictions on number of arrivals from high-risk countries. Once in country, stringent testing and quarantine requirements can be imposed. Countries should also avoid relaxing both travel restrictions and domestic community restrictions at the same time, in order to keep overall community transmission risks low (i.e. maintain R < 1).

The wave of infections experienced by many countries exiting from lockdown highlights the importance of trade-offs when resuming social activities. In the absence of long-lasting herd immunity provided by widespread use of a COVID-19 vaccine, any society’s attempt to return to normalcy with consequent increase in social interactions will need to be carefully calibrated in order to achieve maximum economic and social gains whilst minimizing the cost to health and lives. Policy makers should consider this risk score approach when making decisions about what activities should be resumed, investment in containment and testing capabilities, and building surge capacities.

## Supplementary Material

Risk_budget_paper_for_journal_of_travel_medicine_7_Jul_2021_supp_info_taab113
