# Supplementary material for: Risk scorecard to minimize impact of COVID-19 when reopening
Source: J Travel Med. 2021 Jul 23;28(7):taab113. doi: 10.1093/jtm/taab113 (PMC8420627; doi:10.1093/jtm/taab113)
Supplement: Risk_budget_paper_for_journal_of_travel_medicine_7_Jul_2021_supp_info_taab113 [file risk_budget_paper_for_journal_of_travel_medicine_7_jul_2021_supp_info_taab113.docx]

**SUPPLEMENTARY INFO**

Details on calculation of the various parameters are stated below.

***Determining the contributions of a population’s activities to the budget***

| Observed clinical attack proportion in the household setting (S_household_) | 0.18  Using an observed attack rate in the household setting of 6% and average number of people in a household of 3, based on local outbreak investigation data |
| --- | --- |
| Observed clinical attack proportion in the workplace setting (S_workplace_) | 0.2  Using an observed attack rate at the workplace setting of 2% and average number of close contacts at the workplace of 10 (given prevailing safe distancing measures), based on local outbreak investigation data |
| Probability of one random person appearing at the event ${(P\left( appear \right)}_{activity})$ | $=\frac{Event size}{Population size}$  Population size = 5,000,000^10^ |
| Baseline probability of infection per contact, i.e. talking without wearing mask (${prob(infection)}_{baseline}$) | 0.02  Using an observed attack rate in the social setting of 2%, based on local outbreak investigation data |
| Risk of transmission when engaged in an activity relative to the baseline activity of talking without wearing mask (${relative risk}_{activity}$ ) | Singing / loud talking: 20  Based on the secondary attack rates at choirs of 33%^24^ - 53.3%^25^ (using an approximate number of 40%) divided by the baseline attack rate of 2%  Meetings / conference: 5  Based on the secondary attack rate at a conference cluster in Singapore of 10% divided by the baseline attack rate of 2%  Mask wearing = 0.0225  Based on a systemic review and meta-analysis showing that face mask reduces the probability of infection or transmission by 85%^26^ (i.e. Odds Ratio = 0.15). Assuming both parties (infected individual and the close contact) wear a mask, the odds ratio is 0.15 x 0.15 |
| Estimated number of close contacts (${contacts}_{activity})$ | This was the estimated number of close contacts for each activity based on prevailing safe distancing measures |
| Mean number of secondary infections arising from a single case attending an activity (S_activity_) | $={P\left( appear \right)}_{activity} \times{P\left( infection \right)}_{baseline}\times{relative risk}_{activity}\times{contacts}_{activity}$ |
| Overall mean number of secondary infections arising from the activities undertaken by a population | $S_{overall}= S_{household}+ S_{workplace}+ \sum_{activity} S_{activity}.$ |

***Determining the weekly incidence***

The number of cases generated in the community in week $w$, $C_{comm,w}$, is as follows:

$$C_{comm,w}= S_{overall} \times\left[ \begin{aligned} \epsilon_{imported} n_{import}C_{import,w-1}+ \\ \left( 1-n_{import} \right)C_{import,w-1}+ \\ \epsilon_{comm} n_{comm} C_{comm,w-1}+ \\ \left( 1-n_{comm} \right)C_{comm,w-1}+ \\ C_{superspreading,w-1} \end{aligned} \right].$$

We denote $C_{import}$ as the number of imported cases per week and $n_{import}$ and $n_{comm}$ as the probability of an imported case being notified and community case being isolated respectively. We denote $\epsilon_{imported}$and $\epsilon_{comm}$as the proportion of the notified imported and notified community cases who are infectious and have exposure to susceptible contacts respectively.

The first component, $\epsilon_{imported} n_{import}C_{import,w-1}$ , is the number of notified imported cases in the previous week with community exposure. The second component, $\left( 1-n_{import} \right)C_{import,w-1}$, is the number of imported cases that are not subject to quarantine and/or detected by testing on arrival hence exposed to the community and contribute to community transmission. The third component, $\epsilon_{comm} n_{comm} C_{comm,w-1}$, is the number of notified community cases in the previous week with community exposure. The fourth component, $\left( 1-n_{comm} \right)C_{comm,w-1}$, is the number of missed community cases in the previous week. To account for a sudden injection of cases arising from unforeseen superspreading events in week $w-1$ (cases only surface one week later), we introduce $C_{superspreading,w-1}$ as the fifth component.

The total community cases in any given week $w$ is thus the product of $S_{overall}$ and the sum of all five components above. The number of notified cases (i.e. number of cases that will utilise healthcare resources hence should be maintained at an acceptable level) is thus:

$$N_{w}= n_{comm}C_{comm,w}+ n_{import}C_{import,w}.$$
